# Fourier transform infrared spectroscopy detects distinct TAR DNA-binding protein 43 signatures in frontotemporal lobar degeneration

**DOI:** 10.3389/fnins.2025.1649433

**Published:** 2025-12-04

**Authors:** Rodolfo G. Gatto, Oleksandr Gakh, Jordan M. Wilkins, Arenn F. Carlos, Hossam Youssef, Yong Guo, Jennifer L. Whitwell, Keith A. Josephs, Claudia F. Lucchinetti

**Affiliations:** 1 Department of Neurology, Mayo Clinic, Rochester, MN, United States; 2 Department of Radiology, Mayo Clinic, Rochester, MN, United States; 3 Department of Neurology, University of Texas at Austin, Austin, TX, United States

**Keywords:** frontotemporal lobar degeneration, Fourier transform infrared spectroscopy, histopathology, fluorescence microscopy, tau, TDP-43

## Abstract

**Background:**

Frontotemporal lobar degeneration (FTLD) is a leading cause of cognitive impairment in young adults. A major pathological subtype of FTLD is characterized by positive expression of TAR DNA Binding Protein 43 (TDP-43), referred to as FTLD[TDP]. However, techniques that can be utilized to interrogate and distinguish between various subtypes of FTLD are limited. Herein, we evaluated the potential of Fourier transform infrared (FTIR) spectroscopy to inform on the biomolecular changes in FTLD and to discriminate this disease from other non-FTLD cases.

**Methods:**

Histopathologically confirmed cases from FTLD[TDP] and Alzheimer’s disease (AD) autopsy cases were evaluated using FTIR spectroscopy. Formalin-fixed paraffin-embedded (FFPE) brain tissue sections from the superior and medial temporal lobes were obtained from a single control case, an AD case, an FTLD[TDP] case, and a comorbid FTLD[TDP] case that presented with AD pathology (tau and β-amyloid; FTLD[TDP] + AD). All samples were immunostained for pathological forms of tau, β-amyloid, and TDP-43. Myelin was assessed by proteolipid protein staining. Consecutive tissue sections were scanned by FTIR spectroscopy. Spectral maps were manually segmented, matching ten grey matter (GM) and ten white matter (WM) subregions per case for analysis. Peak-area ratios from lipid and amide functional groups as detected by FTIR spectroscopy were quantified and compared.

**Results:**

Relative to the control tissue, both FTLD cases and AD showed increased ratios of amide I/II, α-helix/unordered proteins, α-helix/phosphorylated proteins, and olefinic/lipid content in GM and WM. The α-helix/unordered ratio was significantly different between FTLD cases and AD, while α-helix/unordered and α-helix/phosphorylated ratios differed significantly between FTLD[TDP] and FTLD[TDP] + AD. Across all cases and brain subregions, FTIR spectroscopy-derived amide I/II, olefinic/lipid, and carboxyl/lipid ratios significantly correlated positively with TDP-43 and tau immunoreactivity (*p*-value < 0.05).

**Conclusion:**

Fourier transform infrared spectroscopy of FFPE brain tissue sections from FTLD[TDP] and AD captures disease-specific changes in the composition of proteins, lipids, and secondary structures. These findings suggest that FTIR spectroscopy can serve as a rapid and cost-effective tool for mapping and quantitating biomolecular alterations in FTLD.

## 1 Introduction

Frontotemporal lobar degeneration (FTLD) is a neuropathological term that refers to a group of disorders primarily affecting the frontal and temporal lobes of the brain (Josephs et al., 2011; Cairns et al., 2007). These disorders are clinically and pathologically distinct from Alzheimer’s disease (AD). Frontotemporal lobar degeneration is characterized by pathological heterogeneity and can be classified based on the type of abnormal protein deposits found in neurons and glial cells within grey matter (GM) and white matter (WM) structures of the affected brain regions. One molecular anomaly in FTLD involves abnormal phosphorylation of the microtubule-associated protein tau (referred to here as FTLD[tau]), including cases linked to mutations in the microtubule-associated protein tau gene (MAPT) (Dickson et al., 2011; Boeve and Hutton, 2008; Bodea et al., 2016). Frontotemporal lobar degeneration can also be associated with the phosphorylation of TAR DNA-binding protein 43 (TDP-43), referred to as FTLD[TDP] (de Boer et al., 2020; López-Carbonero et al., 2024; Carlos and Josephs, 2022; Mackenzie et al., 2011; Neumann et al., 2021). While immunohistochemistry (IHC) remains the standard for diagnosing FTLD[TDP], variable biochemical properties of TDP-43 aggregates can complicate detection of the disease. Thus, we aimed to determine if Fourier-transform infrared (FTIR) spectroscopy, which is sensitive to subtle molecular changes in proteins, lipids, carbohydrates, and nucleic acids, could distinguish between FTLD subtypes, AD, and control human brain tissue.

The TDP-43 protein is a highly conserved nuclear RNA/DNA-binding factor involved in regulating RNA processing (Jo et al., 2020). The C-terminal region of TDP-43 influences both degradation and aggregation (Kasu et al., 2018). In FTLD[TDP], concentrations of phosphorylated TDP-43 increases linearly with age in older adults, regardless of clinical dementia status (Carlos et al., 2022). The topography and morphology of TDP-43 inclusions correlate with specific clinical syndromes and genetic mutations, suggesting distinct pathomechanisms. The pathology of FTLD[TDP] is linked to mutations in genes such as GRN, C9orf72, TBK1, and VCP (Neumann et al., 2021). At the molecular level, evidence suggests that the balance between kinase and phosphatase activities is crucial for controlling TDP-43 phosphorylation (Eck et al., 2021). Dysregulation of these processes can lead to an increase in phosphorylated TDP-43 species (Neumann et al., 2009; Zhang et al., 2009), likely contributing to neurotoxicity in neurons and glial cells (Prater et al., 2022). In neuropathological samples, TDP-43 deposits tend to accumulate in the cellular cytoplasm and nucleus, occurring in neurites of neurons and glial cells (Arseni et al., 2023). The TDP-43 deposits can cause neuronal loss in the superficial layers of the frontotemporal cortex (layers II and III), accompanied by gliosis and occasional spongiosis (Bodea et al., 2016). There are five subtypes of FTLD[TDP] (subtypes A, B, C, D, E), which are distinguished by cellular and regional pathological features and by the distribution of protein aggregates (Neumann et al., 2021).

Definitive diagnosis of FTLD[TDP] relies on IHC targeting the phosphorylated form of TDP-43 (Tan et al., 2013). While IHC is cost-effective and widely used, it can be limited by antibody specificity and technical variability (Raab, 2000). Fourier transform infrared spectroscopy is a label-free and non-destructive technique, which provides information on biochemical changes in lipids, proteins, phosphates, and carbohydrates (Behrman-Lay et al., 2015; Larkin, 2011). Recently, FTIR spectroscopy has been used to capture *in vitro* structural changes across different AD-related protein aggregates (Ramachandran, 2017; Zandomeneghi et al., 2004; Gaber et al., 2023; Álvarez-Marimon et al., 2021). For example, Ramachandran used FTIR spectroscopy to study the transformation of disordered tau protein into cross-β-core containing fibrils due to changes in the secondary structures as captured by alterations in the amide I and amide II spectra (Ramachandran, 2017). Zandomeneghi et al. (2004) utilized FTIR spectroscopy to determine if the β-sheets in the core of amyloid fibrils were structurally similar to that of native β-sheet-containing proteins (e.g., transthyretin). The authors determined that the molecular structure of amyloid fibrils was unique from native β-sheets based on differences detected in their amide I spectra. In another study, Gaber and colleagues demonstrated in murine retinal tissue an association between Aβ42 accumulation and changes throughout the FTIR spectra including the NH-OH, C-H stretching, and fingerprint regions (4000–3000 cm^−1^, 3000–2800 cm^−1^, and 1600–900 cm^−1^, respectively) (Gaber et al., 2023). Although FTIR spectroscopy is gaining traction in the medical field (Rakib et al., 2019; Yonar et al., 2018), and shows promise for ex vivo neuropathological analysis, its widespread validation in FTLD remains limited.

In this study, we evaluated the use of FTIR spectroscopy to analyze formalin-fixed paraffin-embedded (FFPE) brain tissue samples from FTLD[TDP], AD, and control subjects. We hypothesized that FTIR spectroscopy will detect unique biomolecular alterations specific to the various FTLD subtypes and AD compared to control brain tissue. Our results demonstrate unique spectral profiles in different subtypes of FTLD compared to AD and controls, which will aid in the development of FTIR spectroscopy to advance our understanding of molecular changes associated with this disease.

## 2 Materials and methods

### 2.1 Patient population

This study utilized cerebral tissue from the tissue repository at the Mayo Clinic, Rochester, Minnesota, including four participants that were enrolled in an NIH-funded study by the Neurodegenerative Research Group (NRG). Brain autopsies were performed between November 2017 and November 2022. One participant with mild cognitive impairment (MCI) without neuropathological tau or β-amyloid pathology was designated as a control. Two participants with a clinical history of cognitive impairment were diagnosed with FTLD. One case had a progressive history of cognitive decline and was positive for TDP-43 neuropathological depositions (designated as FTLD[TDP]). The FTLD[TDP] case carried the C9ORF72 mutation, which is the most common genetic cause of FTD (Sha et al., 2012; van Blitterswijk et al., 2013). The second case was also with cognitive decline and TDP-43 inclusions with a high degree of AD pathology (i.e., positive for paired helical filament (PHF) tau and β-amyloid immunostaining). This second FTLD case was therefore designated as FTLD[TDP] + AD. The study was approved by the Mayo Clinic IRB, and all patients or proxies consented to the research study. (The proxies provided consent for patients when needed.) The study followed the ethical standards of the Committee on Human Experimentation at Mayo Clinic by the Helsinki Declaration of 197. Demographics of each subject in this study, including the degree of Alzheimer’s disease neuropathologic change (ADNC), are summarized in Table 1.

**Table 1:** Patient demographics.

Subject #	Dx	Age at autopsy	Sex	Brain region	Braak stage	Thal phase	CERAD score	ADNC score	Neuropathological findings
1	CONTROL	84 yo	M	S/M temporal lobe (L)	0	0	0	A0, B0, C0	Not tau or TDP-43 lesions - SEVERE arteriosclerosis
2	FTLD[TDP]	85 yo	M	S/M temporal lobe (L)	II	II	0	A1, B1, C0	TDP-43 lesions and scarce NFTs *
3	FTLD [TDP] + AD	73 yo	F	S/M temporal lobe (R)	VI	V	III	A3, B3, C3	TDP-43 inclusions/extensive tau aggregates/beta-amyloid depositions
4	AD	78 yo	M	S/M temporal lobe (R)	VI	IV	III	A3, B3, C3	Extensive tau aggregates and beta-amyloid depositions

* Patient with C9OR72 mutation. FTLD, frontotemporal lobar dementia; AD, Alzheimer’s disease; L, left; R, right; M, male; F, female; S/M, superior and middle; NFT, neurofilament tangles; ADNC, Alzheimer’s disease neuropathological changes.

### 2.2 Histopathology

#### 2.2.1 Immunohistochemistry procedures

Histochemical processing of paraffin-embedded slides from superior and medial temporal brain block regions was performed using standard procedures described elsewhere (Gatto et al., 2023). Pathological tau burden was detected by 3,3′-diaminobenzidine (DAB) staining using a mouse-host monoclonal phospho-PHF-tau pSer202 + Thr205 antibody (Clone AT8, Thermo Fisher Scientific, MN1020, 1:100) (Bramblett et al., 1993; Brion et al., 1993; Goedert et al., 1995). TAR DNA-binding protein 43 pathology was detected using an antibody recognizing phosphorylated full-length TDP-43 (45KDa) and its C-terminal fragments (Cosmo Bio, TIP-PTD-M01, mouse monoclonal 1:1000) (Josephs et al., 2019). The amino-terminal of β-amyloid was detected with the monoclonal mouse clone 6F/3D antibody (Dako, M0872, mouse monoclonal, 1:50) (Wegiel et al., 2007). Myelin proteolipid protein (PLP) staining (Serotec, Oxford, United Kingdom, 1:500) was performed by standard DAB staining procedures as previously described (Guo et al., 2017). Nuclear counterstaining was performed with hematoxylin. Histological imaging was acquired using a digital pathology microscope scanner (Grundium Ocus 40, Germany). For quantitative measurement, the area of each slide was divided using a standardized grid setting with ImageJ software (Schneider et al., 2012). Ten randomly selected areas centered along GM and WM regions of interest (ROIs) were manually segmented for each brain tissue section (Supplementary Figure 1). Quantitative histological analysis was performed in ImageJ using threshold and masking methods (Jensen, 2013). Each ROI was averaged to obtain a final mean % area value per slide using standard procedures described in previous work (Gatto et al., 2023).

#### 2.2.2 Fluorescence immunohistochemistry analysis

To improve consistency in the characterization of different phosphorylated tau species among different histopathological techniques, we included immunofluorescence (IF) staining for AT8 and TDP-43. Tissue was sectioned at 10 μm and placed onto glass slides followed by standard deparaffinization with xylene. The samples were incubated with lipofuscin autofluorescence quencher (TrueBlack, Biotium, #23007), as previously described (Gatto et al., 2024). Samples were stained using anti-phospho tau (Thermo-Fisher, MN1020, mouse monoclonal, 1:250) that detects AT8 clone of phosphorylated tau (pS202, pT205). The TDP-43 staining was performed using an anti-phospho-TDP (pS409/410) antibody (Cosmo Bio, TIP-PTD-M01, mouse monoclonal, 1:1000). The secondary antibody used was Alexa Fluor 594, Goat anti-mouse, A-11032, 1:200. Slides were mounted with antifade mounting medium containing DAPI (Vectashield plus) for nuclear counterstaining. Imaging was performed by confocal microscopy (LSM 780, Zeiss) and analysis on ImageJ (Jensen, 2013).

### 2.3 Fourier transform infrared spectroscopy

#### 2.3.1 FTIR tissue preparation and data acquisition

Consecutive FFPE brain tissue sections (5 μm thick, supratentorial region) were mounted on BaF_2_ slides (Alkor Technologies, Saint Petersburg, Russia). Tissue sections were deparaffinized by incubating at 65 °C followed by standard xylene/ethanol washes. The FTIR spectra were collected using an Agilent FTIR Cary 620/670 system in transmission mode at a spectral resolution of 8 cm^−1^ and spatial resolution of 20 μm, spanning the 3900–950 cm^−1^ region. The 128 × 128 focal plane array detector was used to record FTIR chemical images. Spectral data were collected with accumulations of 48 scans per pixel. Background spectra were collected from a clean area of each BaF_2_ slide used to compensate for atmospheric and beam current changes. The microscope stage and spectrometer were constantly purged with nitrogen gas. Resolutions Pro software (version 5.2) was used for image generation and collection of all spectral data.

#### 2.3.2 FTIR spectra analysis

All spectra were pre-processed using the open-source software package Quasar (Bioinformatics Laboratory of the University of Ljubljana, Version 0.4.9, Ljubljana, Slovenia) with the spectroscopy add-on (Version 0.4.9) (Toplak et al., 2017; Toplak et al., 2021). Spectral regions corresponding to the CO_2_ band at 2250–2400 cm^−1^ were excluded, and spectra were baseline corrected using the rubber-band method. To compare immunohistochemical stains with FTIR spectroscopy-derived images, ten ROIs from the GM and WM were randomly selected and matched between corresponding samples (please see Supplementary Figure 1). Each ROI consists of approximately 1,000 pixels with each pixel containing a full spectral profile spanning the wavenumbers 3900–950 cm^−1^. The spectra were averaged to generate a representative profile for each ROI to help reduce noise and improve reproducibility. The abundance of various molecular features for each ROI was determined using the area under the absorbance curve. To account for variations in the tissue, we also calculated the ratios among different functional groups (Gakh et al., 2024). Second derivative spectra were calculated using the Savitsky–Golay algorithm (polynomial order 2, window size 5 points), followed by vector normalization. As FTIR spectroscopy measures bulk molecular absorbance containing spectra from mixed protein features, we used adjacent serial sections stained for tau, Aβ, and TDP-43 for correlation analyses.

### 2.4 Statistical analysis

Statistical analysis was performed using GraphPad Prism (version 10.0.0, Boston, Massachusetts, United States) and R (version 4.4.0) software. Global correlative trends were analyzed using all data irrespective of grouping. Spearman’s correlation was used to identify significant trends. Similarly, Spearman’s correlations at a per group basis were performed in R using the package GGally (version 2.2.1). All remaining statistical analyses were performed using the Mann–Whitney test in GraphPad. For all statistical tests, *p*-values lower than 0.05 were considered significant.

## 3 Results

### 3.1 Tau, β-amyloid, and TDP-43 immunohistology confirms type-A FTLD[TDP] and FTLD[TDP] + AD co-pathology

**Figure 1:**
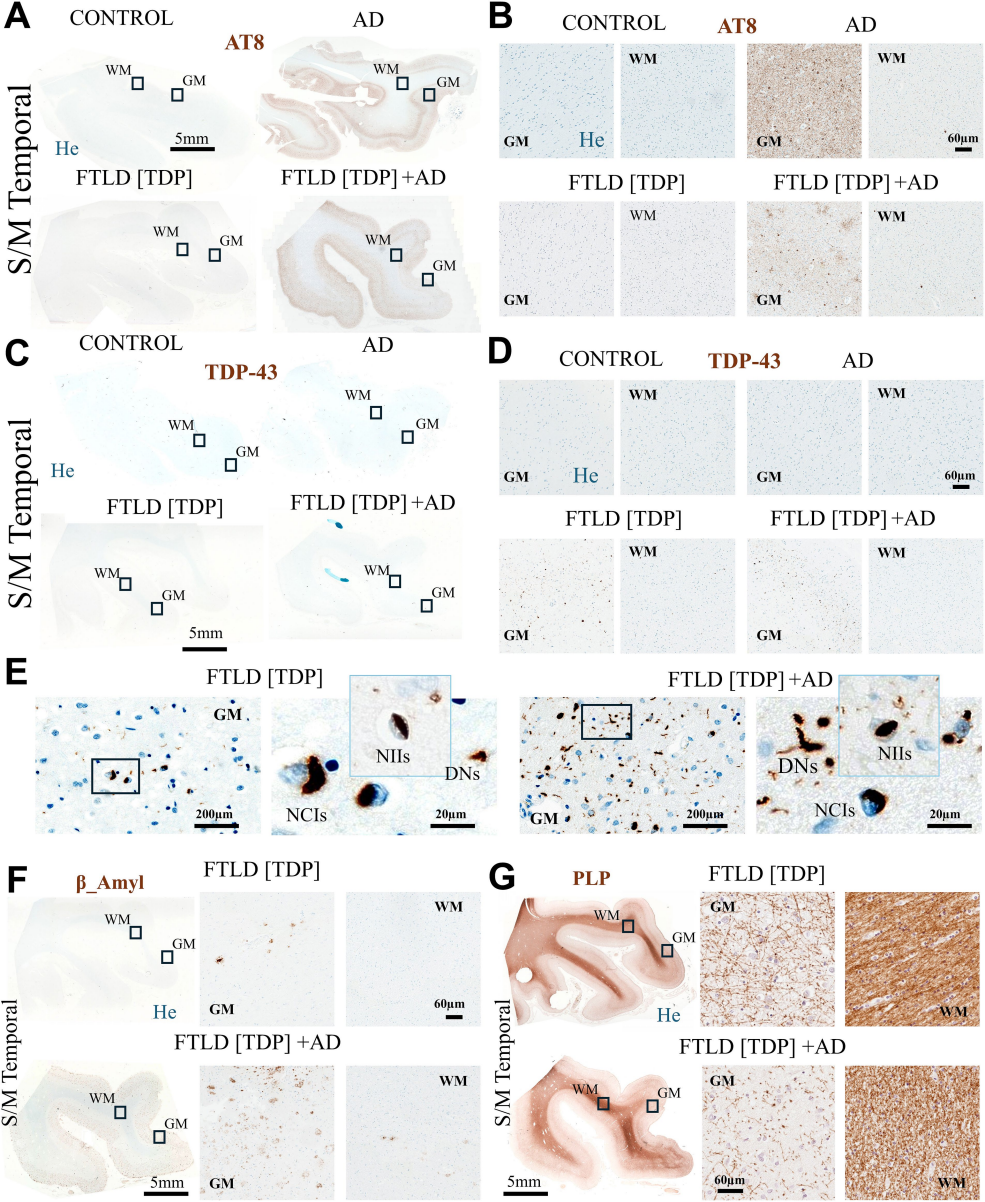
Neuropathological findings across study subjects. **(A)** Staining with AT8 (Tau) across superior and middle temporal samples from each study subject (left panel). **(B)** Higher magnification AT8 staining (right panel) from representative grey and white matter regions, indicated by squares in the left panel. **(C)** Immunohistochemical staining with TDP-43 across the preparations from all study subjects (left panel). **(D)** Corresponding magnification of grey and white matter regions (right panel), indicated by squares in the left panel. **(E)** Additional magnification and morphological features of TDP-43 aggregates in GM regions from FTLD subjects. Additional whole-section and high magnification images of grey and white matter stained for β-amyloid **(F)** and proteolipid protein (PLP) **(G)** are presented for each frontotemporal lobar dementia case. FTLD, frontotemporal lobar dementia; H-AD, high likelihood Alzheimer’s disease; AT8, phosphorylated tau protein; He, hematoxylin; GM, grey matter; WM, white matter; TDP-43, TAR DNA-binding protein 43; PLP, proteolipid protein. NIIs, intranuclear inclusion; NCIs, neuronal cytoplasmic inclusions; DNs, dystrophic neurites.

To verify the diagnostic category of each case, we first established the distribution of hallmark proteinopathies including TDP-43, tau, and β-amyloid (Figure 1). Immunohistology of the superior and medial temporal lobes showed pathological tau (AT8-positive) staining predominantly in the GM of the FTLD[TDP] + AD and AD cases (Figures 1A,B). In contrast, no tau immunoreactivity was observed in the GM of the FTLD[TDP] and control subject (Figures 1A,B). In the GM regions of both FTLD subjects, TDP-43 staining was evident (Figures 1C,D). The FTLD[TDP] case displayed extensive intraneuronal cytoplasmic inclusions (NCIs), neuronal intranuclear inclusions (NIIs), and dystrophic neurites (DNs) (Figure 1E). In the FTLD [TDP] + AD subject, immunoreactive lesions with frequent NCIs and DNs were also observed (Figure 1E). Both FTLD[TDP] and FTLD[TDP] + AD subjects were classified as Type A based on the Harmonized classification system, which categorizes TDP-43 pathology (Mackenzie et al., 2011). Scattered diffuse amyloid plaques were detected in the cortex of the FTLD[TDP] case, whereas abundant neuritic plaques were found in GM and WM regions of the FTLD[TDP] + AD subject (Figure 1F). Compared to FTLD[TDP], a significant decrease in the abundance of myelin was observed in the GM of the FTLD[TDP] + AD case (Figure 1G). Additionally, the organization of myelin appeared irregular in FTLD[TDP] + AD when compared to FTLD[TDP] (Figure 1G). These findings confirm the anticipated proteinopathy profiles for each case.

**Figure 2:**
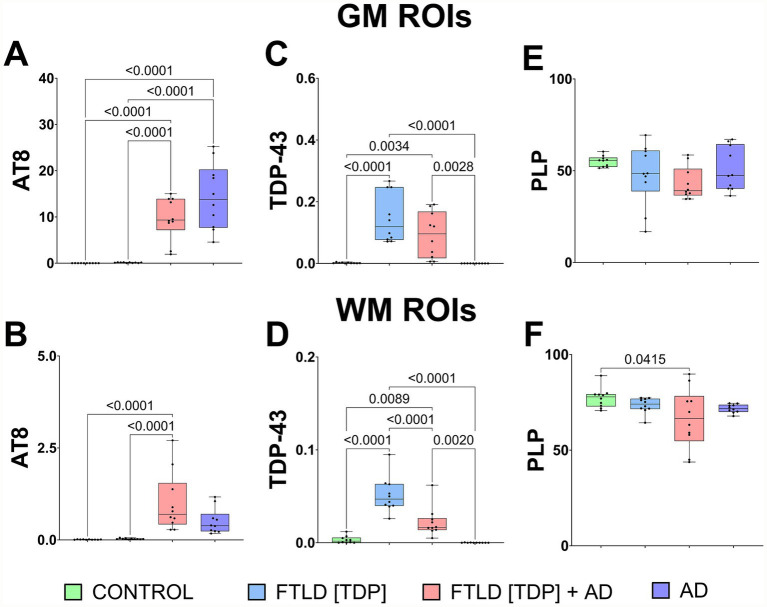
Quantitative analysis of immunohistochemistry from histopathology preparations. Quantitative analysis (% of the area) of AT8 **(A,B)**, TDP-43 stainings **(C,D)**, and PLP **(E,F)** are assessed across 10 randomly assigned grey matter (GM) and white matter (WM) regions from each sample, showing the distribution of each marker.

To look for significant differences between the various proteinopathies, we performed quantitative analysis of the IHC images seen in Figure 1. Quantitative analysis confirms a significant increase of tau in AD and FTLD[TDP] + AD when compared to the control in the GM brain tissue (Figure 2A). In the WM, only FTLD[TDP] + AD had significantly more tau when compared to control (Figure 2B). The levels of TDP-43 in both the GM and WM were significantly higher in the FTLD cases when compared to AD and control samples (Figures 2C,D). Although a significant decrease in PLP was detected in FTLD[TDP] + AD case when compared to the control, the levels of myelin remained relatively consistent across all samples (Figures 2E,F). These results further confirmed the expected widespread inclusion of TDP-43 in the FTLD cases, with abundant tau pathology in the FTLD[TDP] + AD and AD cases.

**Figure 3:**
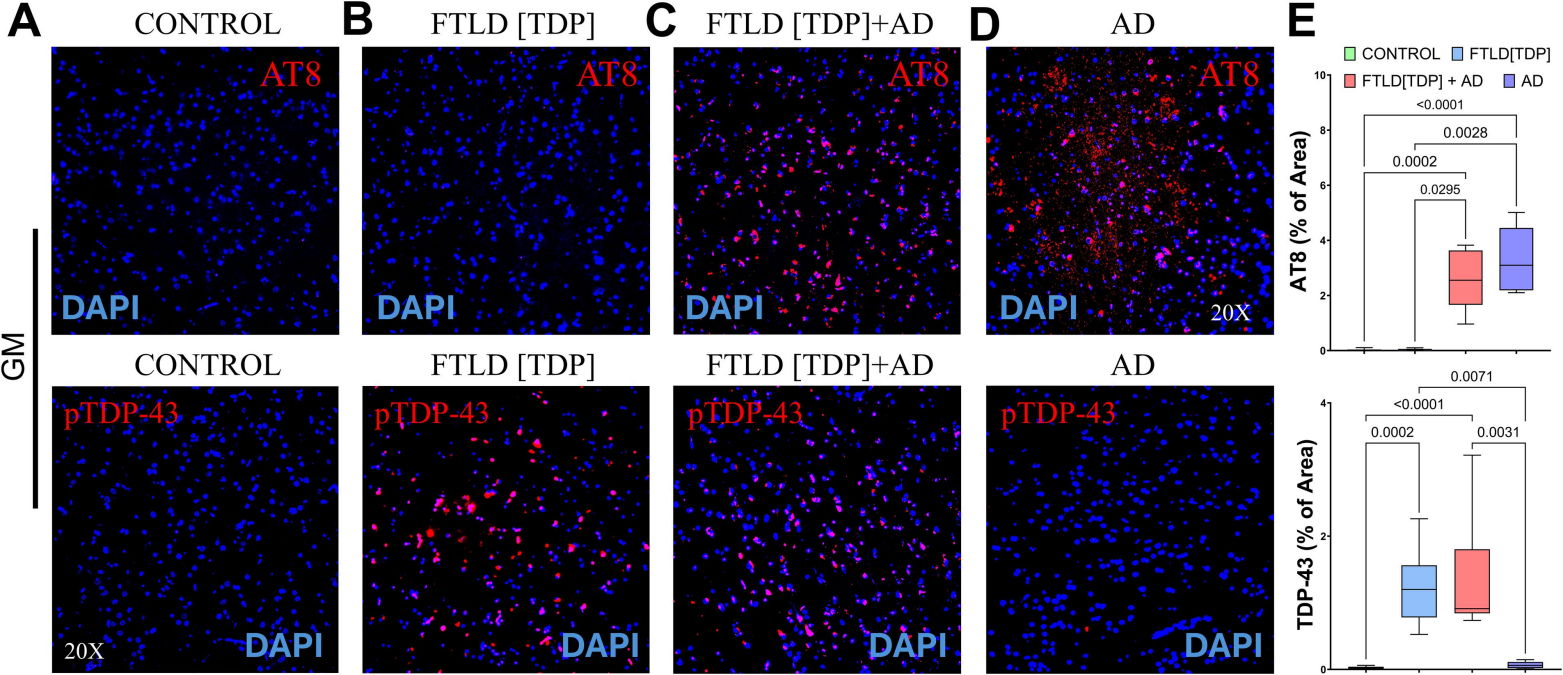
Fluorescence microscopy of control, frontotemporal lobar dementia and AD cases. **(A)** Fluorescence staining of temporal GM regions of control showing negligible signals for TDP-43 and phosphorylated Tau (AT8). **(B)** Temporal GM regions from an FTLD [TDP] case showing positive TDP-43 staining and minimal AT8 signal, consistent with previous DAB staining results. **(C)** Fluorescence IHC staining of an FTLD [TDP] + AD case showing a significant cortical signal for both TDP-43 and AT8. **(D)** AD case showing strong AT8 staining with negligible TDP-43 signal. **(E)** Quantitative analysis of AT8 and TDP-43 stainings from comparable WN and GM ROIs.

Immunofluorescence imaging was used to further confirm the presence of pathological TDP-43 and tau in the control, FTLD cases, and AD subject (Figure 3). Negligible TDP-43 and tau signals were detected in the control GM (Figure 3A). A similar abundance of inclusions of TDP-43 was observed in the GM of both FTLD[TDP] and FTLD[TDP] + AD (Figures 3B,C, respectively). As expected, the levels of tau were decreased in FTLD[TDP] when compared to FTLD[TDP] + AD (Figures 3B,C, respectively). The AD subject had an abundant amount of tau staining with a negligible amount of TDP-43 (Figure 3D). Quantitative analysis from representative GM and WM subregions were calculated (Figure 3E).

### 3.2 Fourier transform infrared spectroscopy reveals unique β-sheet and lipid alterations in FTLD and AD brain tissue

**Figure 4:**
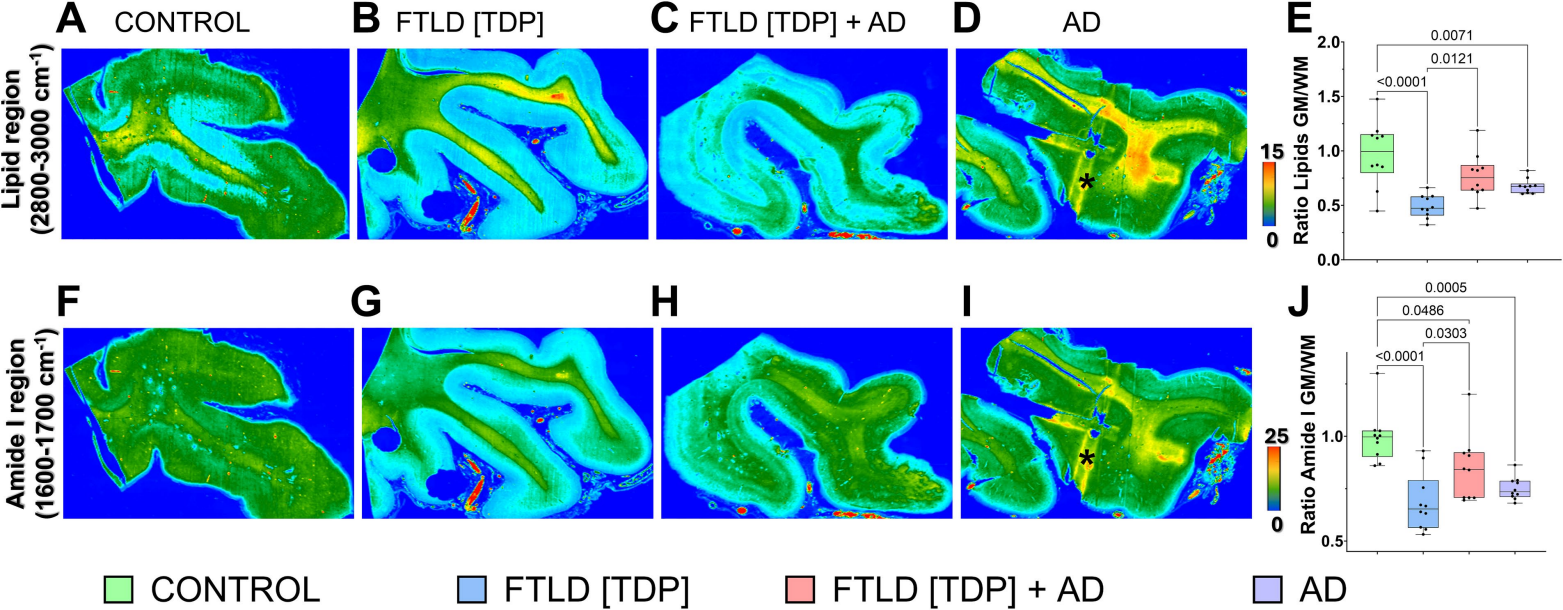
Hyperspectral images show uneven distribution of biomolecules in the brain tissue. Hyperspectral images representing total lipids **(**Panels **A–D)** and Amide I **(**Panels **F–I)** were generated using absorbance intensities from the 2800–3000 cm^−1^ and 1600–1700 cm^−1^ spectral regions, respectively. The panels correspond to the following diagnostic groups **(A,F**: Control; **B,G**: FTLD-TDP; **C,H**: FTLD-TDP + AD; **D,I**: AD**)**. Scale bars indicate relative biomolecular abundance based on absorbance intensity. An asterisk in **(**Panels **D,I)** mark an artifact caused by the plastic grid of the tissue block. **(**Panels **E,J)** show the ratio of lipid and Amide I intensities between gray and white matter regions. Regions of interest (ROIs) used for these calculations are detailed in Supplementary Figure 1.

After defining the histopathological landscape, we applied FTIR spectroscopy and hyperspectral imaging to capture global biomolecular alterations in the brain tissue (Figure 4). The hyperspectral images showed altered amide I (1600–1700 cm^−1^) and lipid (2800–3000 cm^−1^) signals across the GM and WM of the FTLD cases and AD subject compared to the control (Figure 4). Relative changes between the GM and WM lipids show a significant decrease in FTLD[TDP], FTLD[TDP] + AD, and AD compared to the control (Figure 4E). Similarly, we saw significant decreases in GM to WM amide I levels in FTLD[TDP], FTLD[TDP] + AD, and AD compared to the control (Figure 4J). These observations indicated that both lipid and protein pools are altered in FTLD and AD compared to the control.

**Figure 5:**
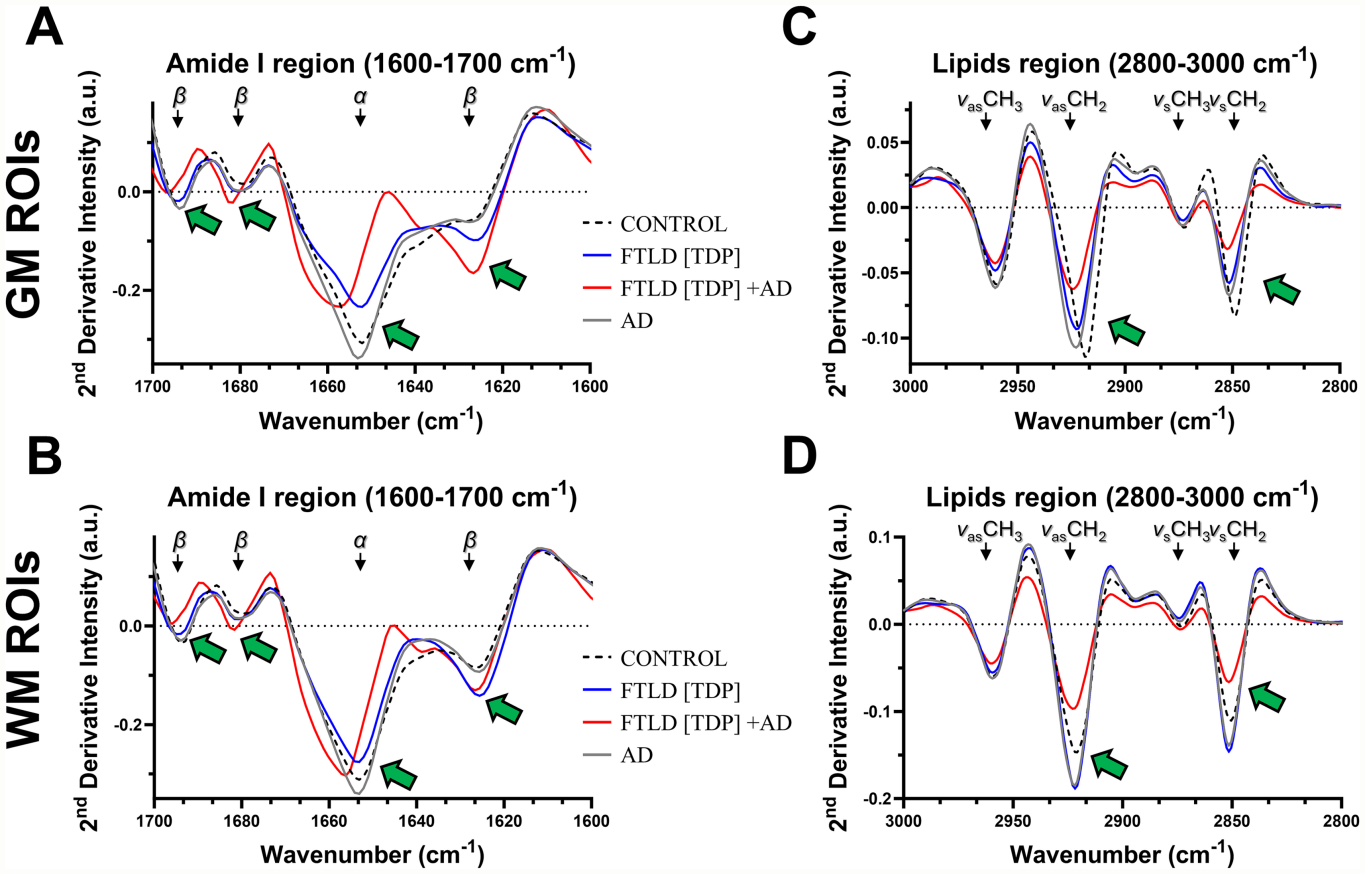
Spectral characterization of neuropathological FTLD samples by FTIR. **(A,B)** FTIR second derivative spectral analysis of FTLD [TDP] and FTLD [TDP] + AD superior and middle temporal region samples across all GM /WM regions showing characteristic patterns of changes in the amide I region (green arrows indicate peak change). **(C,D)** Further spectral evaluation across GM/WM ROIs demonstrated the main spectral differences between FTLD cases present in subregions of the lipids window (green arrows).

Next, we aimed to determine secondary structural changes in the lipids and amides in the brain tissue of the FTLD and AD cases compared to the control (Figure 5). Second derivative spectral analysis from matched ROIs revealed a prominent increase in intensity of the β-sheet peak located near 1625 cm^−1^ in the FTLD cases compared to the AD and control subjects (Figures 5A,B). In the GM, the β-sheet peak at 1625 cm^−1^ was the most intense in FTLD[TDP] + AD compared to FTLD[TPD] (Figure 5A). In the WM, the β-sheet peak was similar in both FTLD cases (Figure 5B). The peak near 1652 cm^−1^ largely represents the *α*-helical structure of amides. In the GM, both FTLD cases had a reduced intensity of the α-helix compared to the AD and control subjects (Figure 5A). Moreso, the FTLD[TDP] + AD case had a shift towards 1660 cm^−1^ compared to all other cases, which was also apparent in the WM (Figures 5A,B). The intensity of the α-helix was more similar across all cases in the WM (Figure 5B). The FTLD[TDP] + AD case displayed additional spectral shifts in the amide region associated with the beta structures near 1625 cm^−1^, 1680 cm^−1^, and 1695 cm^−1^ when compared to FTLD[TDP], AD, and control cases.

In the lipid region (2800–3000 cm^−1^), second derivative analysis showed marked differences between all cases. A decrease in the intensity of the CH_2_ symmetric and asymmetric bands (2850 cm^−1^ and 2920 cm^−1^, respectively) was most prominent in the FTLD[TDP] + AD case compared to FTLD[TDP], AD, and control in both GM and WM (Figures 5C,D). Additionally, both FTLD cases and the AD subject showed a shift towards higher wavenumbers (2923 cm^−1^ vs. 2918 cm^−1^) compared to the control tissue (Figures 5C,D). Taken together, these results point to pronounced β-sheet, α-helical, and lipid chain perturbations in the FTLD cases, particularly in the FTLD[TDP] + AD case, when compared to control tissue.

**Figure 6:**
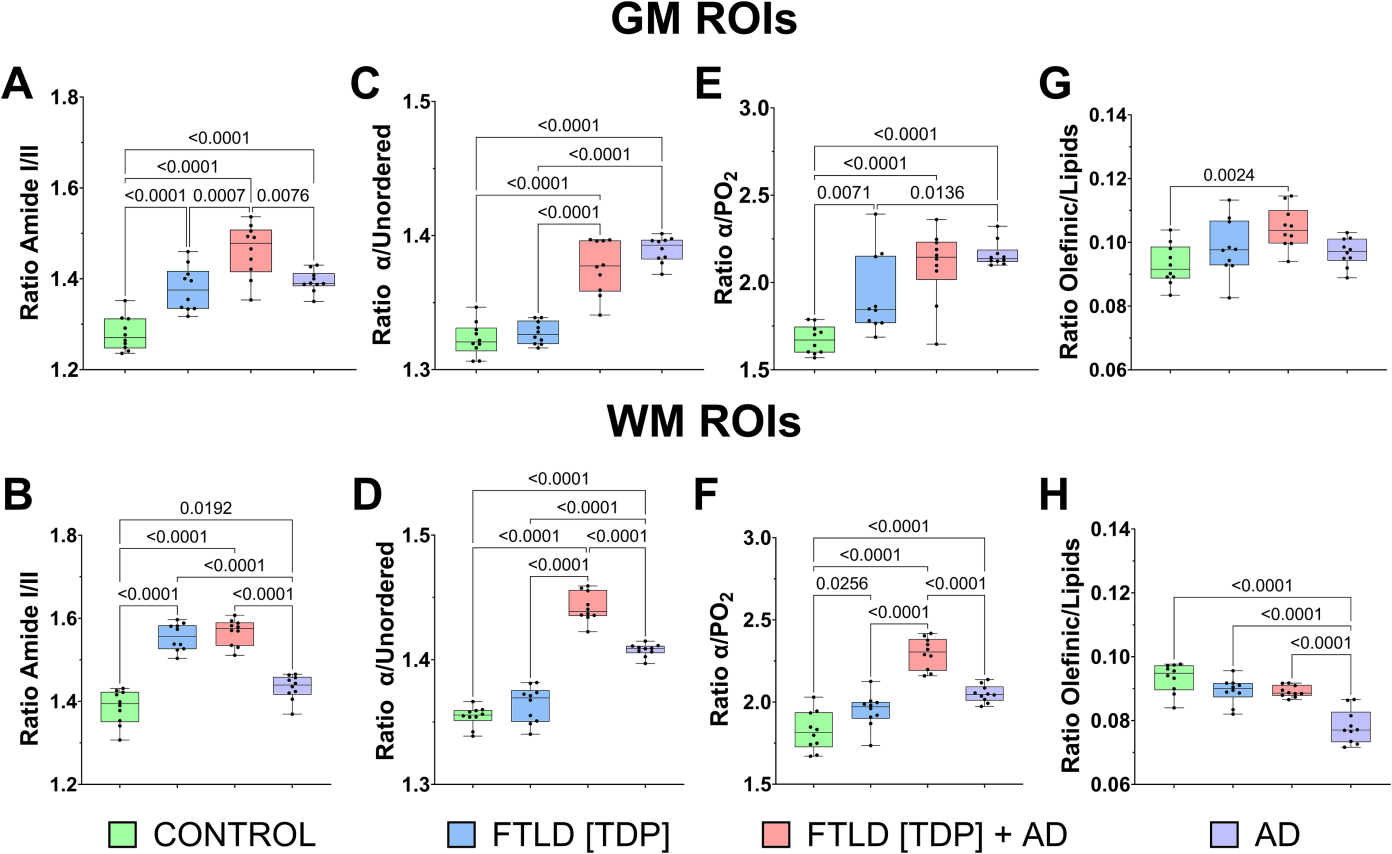
Quantitative evaluation of FTIR markers on neuropathological samples. **(A)** Overall changes of Amid I/II (protein rearrangement), **(C)** alpha/unordered (coil elements), **(E)** alpha/PO_2_ (protein phosphorylation), and **(G)** olefinic/lipid (membrane fluidity/lipid peroxidation) ratios were calculated from GM’s average ROIs for each neuropathological sample. **(B, D, F, H)** Measurements on each FTIR parameter were also performed on average WM areas.

To quantify biomolecular changes, we calculated biologically relevant band ratios that correspond to protein and lipid structure, phosphorylation, and lipid oxidation. The amide I/II ratio was significantly increased in the GM and WM of the FTLD and AD subjects compared to the control (Figures 6A,B). In the GM, the amide I/II ratio was significantly higher in FTLD[TDP] + AD compared to all other cases (Figure 6A). In the WM, both FTLD cases had a significantly higher amide I/II ratio compared to AD and control subjects (Figure 6B). Protein structural changes were, in part, assessed by the α-helix/unordered ratio, which was significantly higher in the FTLD[TDP] + AD and AD cases compared to the control in both the GM and WM (Figures 6C,D). In the WM, the *α*-helix/unordered ratio was significantly increased in the FTLD[TDP] + AD case compared to all other subjects (Figure 6D). The α-helix/PO_2_ ratio, used as a proxy for protein phosphorylation, was significantly increased in both FTLD cases and the AD subject in both GM and WM when compared to the control (Figures 6E,F). In the WM, the α-helix/PO_2_ ratio was significantly higher in the FTLD[TDP] + AD case compared to all other subjects (Figure 6F). Lipid peroxidation was assessed by the olefinic/lipid ratio, which was significantly increased in the GM of the FTLD[TDP] sample compared to the control (Figure 6G). A significant decrease in the olefinic/lipid ratio was seen in the WM of AD tissue compared to all other cases (Figure 6H). The ratio of carboxyl functional groups to total lipids, another indicator of lipid oxidation, further showed a significant increase in the GM of FTLD[TDP] + AD, and FTLD[TDP], compared to the control and AD samples (Supplementary Figure 2). However, in contrast to the olefinic/lipids ratio, the carboxyl/lipids ratio in the WM showed significant increases in FTLD[TDP] + AD and FTLD[TDP] when compared to AD and control samples.

Overall, FTIR spectroscopy offers the opportunity to quantifiably separate FTLD[TDP] subtypes from other diseases and control brain tissue. Our findings provide insight into changes in β-sheets, phosphorylation, lipid and protein structure, and lipid oxidation, which may aid in the development of biochemical fingerprints for FTLD reflecting underlying pathophysiological mechanisms.

### 3.3 Tau and TDP-43 burden correlate with biomolecular changes detected by FTIR spectroscopy

**Figure 7:**
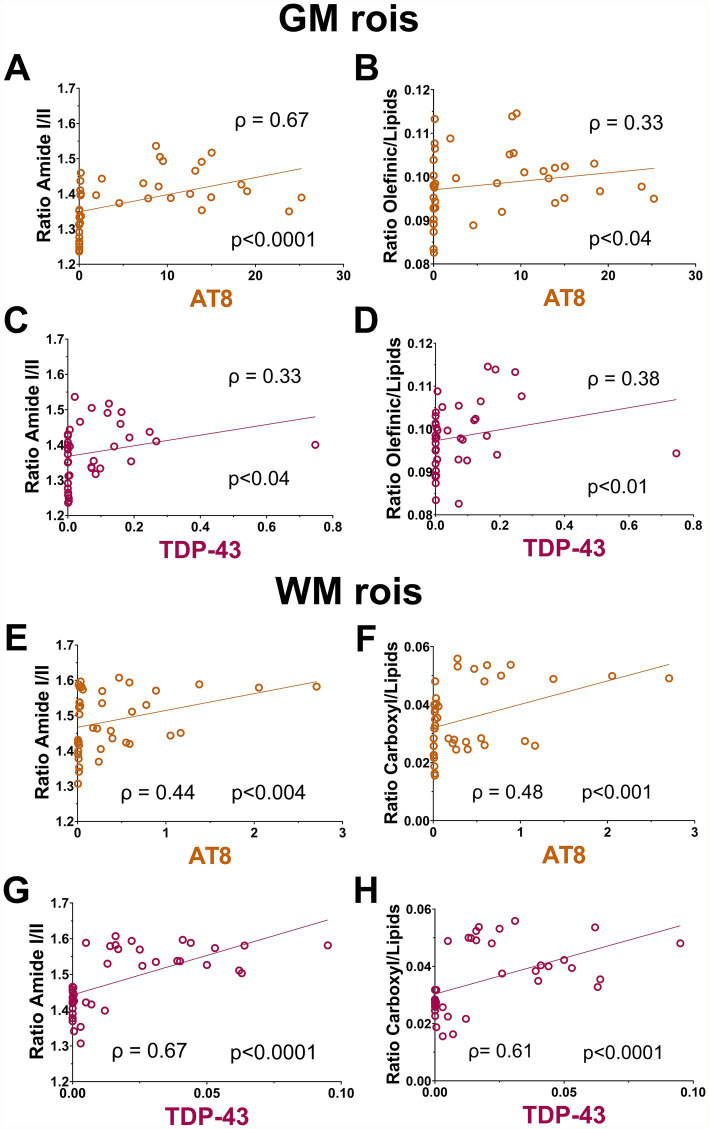
Correlative measurements between histo and FTIR. **(A–D)** Significant correlations between AT8 (TAU) and TFDP-43 and Amid I/II and Olefinic/lipid ratios can be measured across GM areas. **(E–H)** Significant correlative changes across neuropathological and FTIR markers were also seen across WM subregions. Correlations were calculated using data points from matched ROIs from all subjects to determine the overall association between biochemical markers gathered from histopathology and FTIR signals.

To assess if biological ratios measured with FTIR spectroscopy reflected disease severity, we correlated them with tau and TDP-43 burden detected by IHC (Figure 7). First, we utilized all ROI measurements taken across all four cases (control, FTLD[TDP], FTLD[TDP] + AD, and AD). In the GM, the ratios of amide I/II and olefinic/lipids had a significant positive correlation with tau burden (Figures 7A,B, respectively). Similarly, the amide I/II and olefinic/lipids ratios significantly correlated with TDP-43 burden in the GM (Figures 7C,D, respectively). In the WM, tau had a significant positive correlation with the amide I/II and carboxyl/lipid ratios (Figures 7E,F, respectively). Likewise, these ratios significantly correlated with TDP-43 in the WM (Figures 7G,H, respectively). These observations may suggest that structural and oxidative indices detected by FTIR spectroscopy rise with histological tau and TDP-43 burden.

We then examined correlations within each group to look for disease-specific signatures. In the GM, a significant positive correlation between CH_3_/lipids and α-helix/unordered was observed in the control but absent in the FTLD and AD cases (Supplementary Figure 3A). Amide I had a significant positive correlation with lipids in FTLD[TDP] + AD and AD, which was not observed in FTLD[TDP] and the control (Supplementary Figure 3A). These observations in the GM may highlight disruptions in FTLD and AD cases, some of which may, in part, be associated with tau pathology. In the WM, a significant negative correlation was observed between amide I and *α*-helix/β-sheets only in the FTLD[TDP] case (Supplementary Figure 3B). The α-helix/unordered to α-helix/β-sheets had a significant positive correlation in FTLD[TDP] + AD and FTLD[TDP], which was absent in AD and control (Supplementary Figure 3B). These findings may indicate FTLD specific signatures in WM when compared to AD and control. Taken together, correlation analyses demonstrate that FTIR spectroscopy-derived markers may help distinguish between various subtypes of FTLD from control and AD brain tissue.

## 4 Discussion

In this study, we used FTIR spectroscopy on human brain tissue to identify distinct biomolecular signatures that separate FTLD[TDP], FTLD-TDP-AD, AD, and control individuals. Our findings further demonstrate that unique secondary structural changes exist within each disease group. More so, FTIR spectroscopy-derived indices scale with tau and TDP-43 loads. Taking together, these results show that FTIR spectroscopy is sensitive to both secondary structure changes and lipid disruption in different subtypes of FTLD.

Misfolding of TDP-43 is well characterized in FTLD[TDP], though the molecular features remain under active investigation (Jo et al., 2020; Eck et al., 2021). A previous *in vitro* study associated structural changes in oligomeric TDP-43 fibril assembly and toxicity as detected by several methods including FTIR spectroscopy (Laos et al., 2019). Our FTIR spectroscopy data showed marked alterations in the amide I region of the spectrum, particularly at 1625 cm^−1^ with increased intensity in FTLD[TDP] + AD and FTLD[TDP] compared to AD and control. This peak may reflect altered TDP-43 oligomeric formation in FTLD cases compared to AD and controls possibly reflecting alterations in cytotoxic structures (Laos et al., 2019). Second derivative analysis of FTLD[TDP] + AD spectra highlighted distinct shifts within the amide I band (1600–1700 cm^−1^), which may imply that protein structural alterations and misfolding events occur more frequently. These changes may contribute to the vulnerability of neuronal populations in FTLD (Daniels et al., 2024). Furthermore, we observed significant positive correlations between the amide I/II ratio and histopathological concentrations of TDP-43 when examining data across all cases (control, AD, FTLD[TDP], and FTLD[TDP] + AD). These correlations suggest a potential link between increased protein misfolding and accumulation.

TAR DNA-binding protein 43is known to undergo physiological phosphorylation during its normal function (Nilaver and Urbanski, 2023). In pathological conditions, however, TDP-43 becomes hyperphosphorylated and aggregates due to its intrinsic hydrophobicity and low solubility (Esposto and Martic, 2021). In our study, we detected a significant increase in the *α*-helix/phosphate ratio in FTLD[TDP], FTLD[TDP] + AD, and AD samples across GM and WM when compared to the control. The α-helix/phosphate ratio may reflect contributions from other phosphorylated proteins, including tau. Moreover, our histological findings, support the presence of phosphorylated tau in FTLD[TDP] + AD case. We also observed elevated α-helix /unordered ratios in GM regions of both FTLD[TDP] + AD and AD samples when compared to FTLD[TDP] and control. This pattern may reflect tau or β-amyloid conformational changes rather than exclusively by TDP-43 misfolding. In contrast, the WM distribution of α-helix/unordered ratios was most prominent in the FTLD[TDP] + AD case, possibly indicating a synergistic contribution of both TDP-43 and tau pathologies in these regions. However, given that both tau and β-amyloid contain alpha and beta structural motifs, mixed contributions cannot be excluded, particularly in the FTLD[TDP] + AD and AD cases.

To differentiate between FTLD, AD, and controls, we emphasize that the combination of multiple FTIR-derived ratios and peak shifts, rather than a single marker, is likely needed to be informative. For instance, the α-helix/unordered ratio was notably elevated in both FTLD[TDP] + AD and AD samples compared to FTLD[TDP] and control cases in both the WM and GM. On the other hand, the carboxyl/lipid ratio and β-sheet peak intensity near 1625 cm^−1^ were significantly higher in FTLD[TDP] than AD. Likewise, the FTLD[TDP] + AD case appeared to have unique spectral shits of beta structures near 1625 cm^−1^, 1680 cm^−1^, and 1695 cm^−1^ when compared to FTLD[TDP], AD, and control cases. Thus, a multivariate FTIR signature that integrates multiple indices will likely be required to effectively distinguish FTLD from AD and controls.

The olefinic/lipid ratio was elevated in FTLD[TDP] + AD compared to the control, suggesting possible involvement of membrane degradation or oxidative lipid damage in disease progression. Moreover, the carboxyl/lipid ratio, another index of lipid peroxidation, was elevated in both FTLD[TDP] and FTLD[TDP] + AD samples compared to the control and AD cases. The AD and control cases showed similar carboxyl/lipid levels. Thus, TDP-43 pathology in FTLD may increase lipid oxidation in the brain (Andrés-Benito et al., 2021; Phan et al., 2020; Godoy-Corchuelo et al., 2022). The positive correlation across all samples between carboxyl/lipid ratios and TDP-43 in WM histopathology further strengthens this potential mechanistic link and suggests a broader role for lipid dysregulation in FTLD pathogenesis. While the FTIR-derived olefinic/lipid and carboxyl/lipids ratios are used as indices of lipid peroxidation (Abdelrazzak et al., 2021), we can only infer global changes in the oxidation status via the detection of functional groups including carbonyls (C=O, ~1740 cm^−1^) and olefinic (C=C, ~3010 cm^−1^). Future studies using oxidized lipid species-specific probes (e.g., malondialdehyde (MDA), 4-hydroxynonenal (4-HNE)) will be required to identify precise molecular pathways altered.

Additionally, recent findings indicate that disrupted myelin lipid metabolism differentiates FTLD subtypes caused by *GRN* and *C9orf72* mutations (Marian et al., 2023). For example, TDP-43-GRN compared to FTD-C9orf72 and control cases exhibit increased acylcarnitines in frontal GM and cholesterol ester accumulation in WM, consistent with myelin breakdown (Marian et al., 2023). These changes may help explain the observed association between TDP-43 levels and FTIR spectroscopy-derived lipid ratios in our C9orf72-confirmed FTLD[TDP] case. However, despite these correlations, our quantitative lipid peroxidation ratio (olefinic/lipids) was significantly elevated only in the GM of the FTLD[TDP] + AD sample. On the contrary, the carboxyl/lipid ratio was elevated in the GM and WM in all FTLD samples but not in the AD sample when compared to the control. This finding appears to slightly contradict the FTIR-lipid correlations described earlier and highlights the need for further investigation. It is possible that while FTIR spectroscopy detects biochemical shifts related to lipid unsaturation and oxidation, these do not necessarily align with conventional quantitative peroxidation assays or may vary by region and disease stage.

One of the main limitations of this study is the small sample size, which constrains statistical power and limits generalizability. Another limitation of FTIR spectroscopy is that vibrations from multiple proteins overlap within a single pixel/ROI, precluding direct separation of signals from tau, Aβ, and TDP-43. Our approach used serially adjacent sections to map immunoreactive regions onto corresponding FTIR ROIs, allowing correlation rather than direct spectral decomposition. Emerging FTIR platforms and multimodal microscopy techniques may provide molecular specificity at single-aggregate resolution (Banerjee and Ghosh, 2021; Vosough and Barth, 2021; Cascella et al., 2023). Additionally, the presence of comorbid vascular pathology may result in an altered metabolic state causing changes in the fingerprint region detected by FTIR spectroscopy, which can be sensitive to changes in metabolism and biomolecules (Nazarenko et al., 2017; Mateus Pereira de Souza et al., 2023). In the future, protein standards would help verify spectral changes that were observed here. Although our study did not include spectra from purified proteins (e.g., tau, Aβ, TDP-43), previous studies have interrogated similar changes described here (Zandomeneghi et al., 2004; Laos et al., 2019; Sarroukh et al., 2011; Sadqi et al., 2002). Despite these limitations, our primary aim was to demonstrate the feasibility of ex vivo FTIR spectroscopy in distinguishing spectral profiles between AD and FTLD subtypes and control tissue. However, a key methodological limitation lies in the imprecise coregistration between FTIR data and IHC measurements, likely due to manual alignment of ROIs. This spatial mismatch may underline discrepancies in quantitative comparisons. To improve this, future work will include automated segmentation techniques to increase the accuracy of FTIR-histology alignment. We also plan to expand the cohort with more subjects and brain regions to validate these findings and enhance subtype differentiation in neurodegenerative disease diagnostics.

## 5 Conclusion

This preliminary study underscores the potential of FTIR spectroscopy as a valuable tool for identifying distinct biochemical patterns associated with FTLD, particularly those involving TDP-43 pathology. The observed correlations between FTIR spectral features and neuropathological markers likely reflect shared biochemical disruptions across misfolded proteins and lipid alterations within affected brain regions. By capturing these molecular fingerprints, FTIR offers a promising ex vivo approach for rapid, cost-effective screening that may complement traditional histopathological techniques. With further validation, this methodology could enhance our ability to differentiate neurodegenerative subtypes and provide deeper insights into the biochemical underpinnings of protein misfolding disorders such as FTLD and AD.

## Data Availability

The original contributions presented in the study are included in the article/Supplementary material, further inquiries can be directed to the corresponding authors.
