# Supplementary material for: Fourier transform infrared spectroscopy detects distinct TAR DNA-binding protein 43 signatures in frontotemporal lobar degeneration
Source: Front Neurosci. 2025 Dec 4;19:1649433. doi: 10.3389/fnins.2025.1649433 (PMC12711859; doi:10.3389/fnins.2025.1649433)
Supplement: Supplementary file 2 [file Data_Sheet_2.docx]

***SUPPLEMENTARY MATERIAL***

**Supplementary Figure 2:** FTIR evaluation of carboxyl functional groups /lipids ratios from GM (**A**) and WM (**B**) regions across all specimens.
