# Supplementary material for: Fourier transform infrared spectroscopy detects distinct TAR DNA-binding protein 43 signatures in frontotemporal lobar degeneration
Source: Front Neurosci. 2025 Dec 4;19:1649433. doi: 10.3389/fnins.2025.1649433 (PMC12711859; doi:10.3389/fnins.2025.1649433)
Supplement: Supplementary file 3 [file Data_Sheet_3.docx]

***SUPPLEMENTARY MATERIAL***


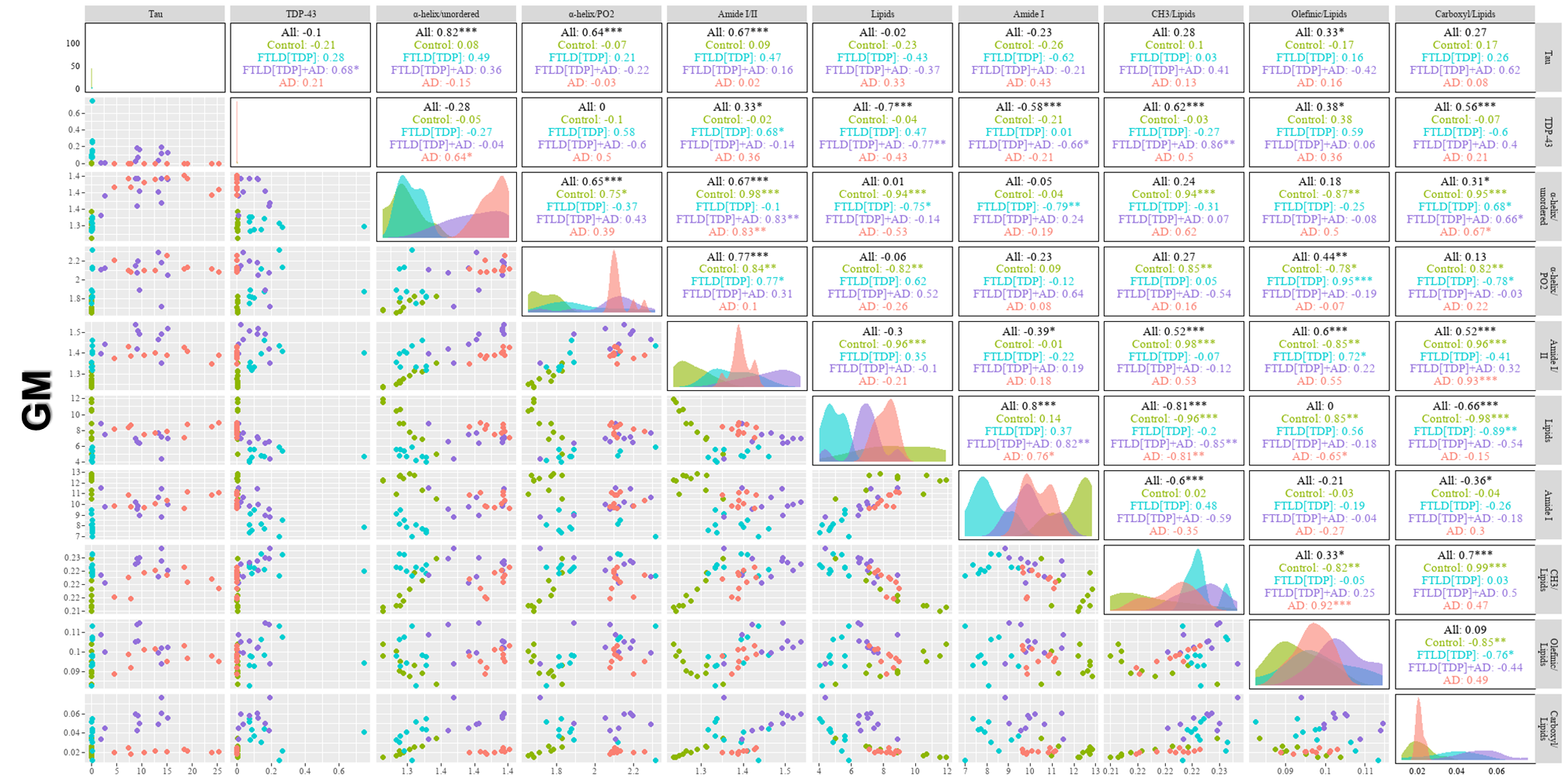
**A**

**
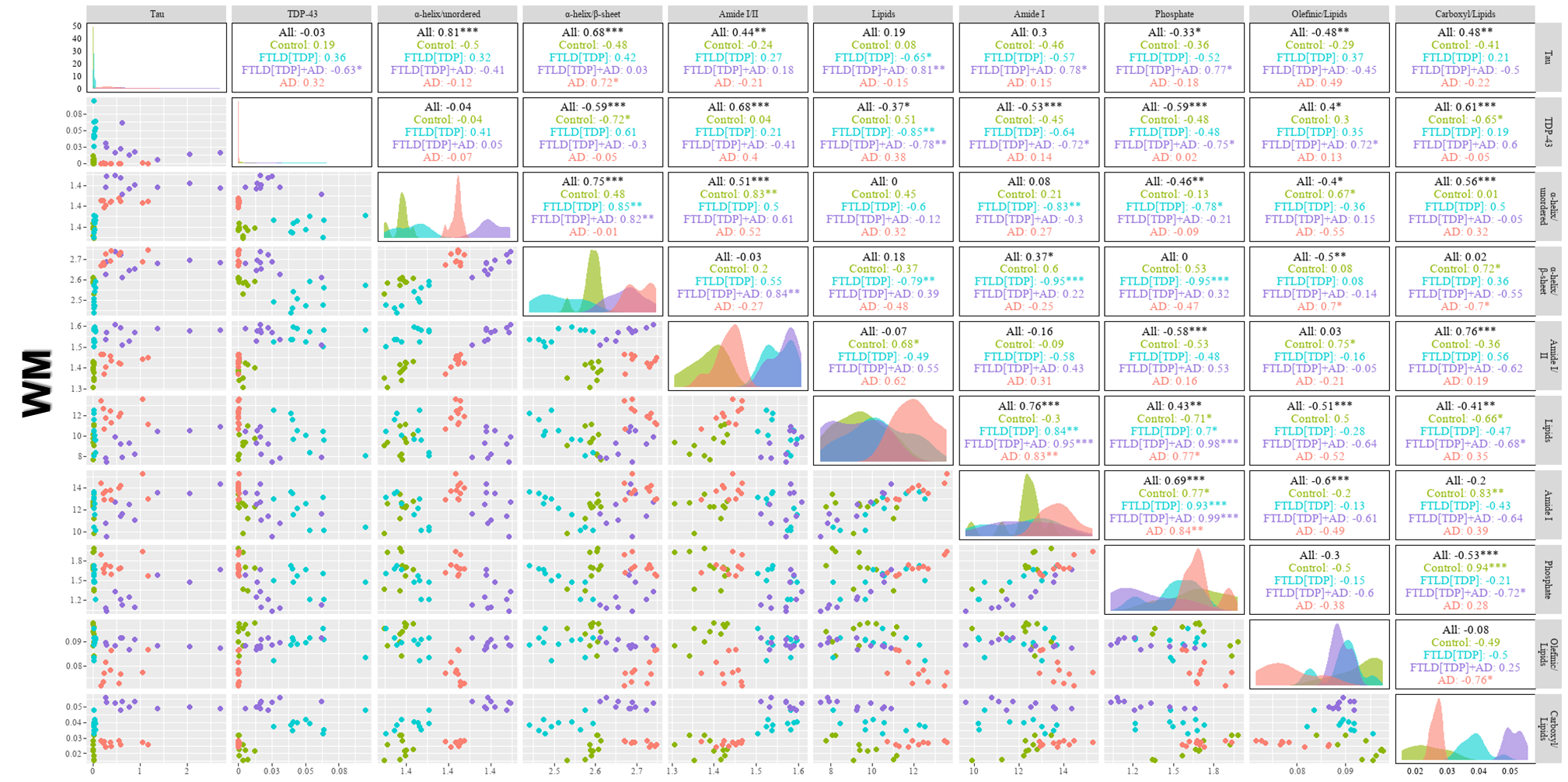
B**

**Supplementary Figure 3:** Additional correlations between histopathological and FTIR parameters considered per subject. Cross-

correlation plots using Spearman’s correlation coefficients methods obtained from cortical **(A**) and white matter **(B**) ROIs. Overall correlations (black ink), and individual correlations from the control (green), AD (orange), FTLD[TDP] (blue), and FTLD[TDP]+AD (purple) subjects are presented.
